# Force prediction in the cylindrical grip for a model of hand prosthesis

**DOI:** 10.1038/s41598-023-43600-1

**Published:** 2023-10-11

**Authors:** Ewelina Drelich, Jan Tracz, Adam Cisowski, Michał Kowalik, Aleksy Figurski, Monika Kwacz, Witold Rządkowski

**Affiliations:** 1 Institute of Micromechanics and Photonics, Warsaw University of Technology, Św. Andrzeja Boboli 8, 02-525 Warsaw, Poland; 2 Institute of Aeronautics and Applied Mechanics, Warsaw University of Technology, Nowowiejska 24, 00-665 Warsaw, Poland

**Keywords:** Biomedical engineering, Applied mathematics

## Abstract

The aim of this paper is to present an analytical method of calculating forces acting on the thumb, index, middle finger, and metacarpal part of a hand prosthesis in a cylindrical grip. This prehension pattern represents a common operation of grabbing and manipulating everyday life objects. The design process assumed that such a prosthesis would have 5 fully operating fingers and 18 total degrees of freedom: three for each finger including the thumb, and another three for the wrist. The assumed load was 1 kg and the diameter equaled 70 mm, representing a water bottle. The method was based on analytical mechanics and as opposed to experiments or numerical methods does not require many resources. The calculations involved solving a system with seven unknown forces using an equilibrium equation for forces and moments in all three axes. The resulting equations were presented in a matrix form and solved using MATLAB software. The validation of the method with an experiment using FSR sensors and comparing it to other reports showed differences in index and middle finger involvement. However, the total sum of forces was similar, therefore it is reasoned that the grip can be performed and the prediction was accurate for the thumb and metacarpal. When using the model, the friction coefficient must be chosen with a safe margin as it influences the grip force. The presented method can be used for other models and designs by inserting their dimensions into the equations and solving them numerically to obtain forces useful in mechatronics design.

## Introduction

The human hand is considered one of the most complicated mechanisms. Its almost unlimited functionality i.e., the ability to grip, manipulate objects, and make gestures is possible due to numerous degrees of freedom and characteristic reverse-oriented thumb. These unique features made it a scope of scientific research with the goal to understand its functionality. It is also known that with aging functionality decreases and what follows—the ability to perform many kinds of labor including everyday tasks such as preparing food cleaning, taking medications, manipulating objects, or making gestures. Even more burdensome in everyday functioning is the case of the lack or loss of the upper limb or its part. Although the surgical removal of a limb is performed only when the patient's health and life are at risk, or when it is not possible to restore the limb's function. In the United States alone there were 1.7 M people with limb loss, which accounted for about 1 in 200 people. Loss of upper extremity is less frequent as it affects about 41,000 persons, which is about 3% of amputees in the US^1^.

As a result of the Afghanistan and Iraq conflicts, there was a need to amputate at least one of the hands in the case of 1558 American soldiers^2^. Amputation is also a necessity in case of severe infection, a traumatic injury that restricts normal functioning, IV-degree frostbite as well as deep damage of bone tissue because of cancer. The most common disease causing the need to perform amputation is diabetes. In the years 1988–2009 the number of amputations related to this disease has risen by 24%^3,4^.

The current market offers many kinds of prostheses, which differ in functionality, and thus are priced differently. It is important to underline the fact that the group that is at the highest risk of amputation are patients, that are in the lower social class. This makes it impossible for them to purchase commercial prosthesis, as the cost of body powered prosthesis may cost even be up to $10,000^5^ and externally powered ones reach $75,000^6^. Although recently the costs have decreased as Open bionic prosthetics Hero Arm^7^ became available with a pricing of $3,000 and the True Limb^8^ device costs $8000 for adults and $4000 for children. There are also worldwide communities such as e-nable that accelerated the availability of 3-D printed prosthetics by gathering volunteers involved with the field^9^. Also, it is worth to mention one of the first 3d-printed prosthetics from 2012 called, Robohand, which has probably inspired the movement^10^. The review of upper limb prosthetics from 2017 showed that there are over 50 3D-printable devices, where the cost of materials is below $500^11^. Application of lightweight materials^12^ and 3D printing methods to print the skeletal structure^13^ of a hand^14,15^ reduced the cost associated with purchasing a new prosthesis, at the same time adopting it to the individual needs of a patient.

The above data suggest a need for anthropomorphic prosthesis, particularly hand prosthetics, which would enable a return to normal functioning in society. Another important fact is, excluding children born without a limb, the prosthesis process should begin as soon as possible after amputation. It is due to the fact that, there exists a possibility of a patient adapting to the new reality, making it difficult to adapt to the prosthesis. Infants with an inborn flaw should obtain the artificial limb at the age, at which they begin to crawl and walk. Such prosthesis would enable them to move and help with grabbing and holding objects^16^. All the above indicates how important it is to create fully functional, easy access, and possibly anthropomorphic prosthesis, which will help the amputees to recover independence in everyday tasks as well as grow in other areas.

The design of such devices was presented in studies that focus on various engineering aspects of the devices. Designers must consider many things, such as using or reusing the existing prosthetic sockets or designing a new one^17^. Other considerations include the number of digits and the number of electromyography signals that can be obtained. The wrist can also be made functional as opposed to other designs that don’t include such a solution^18^. The manufacturing technology available to the designer is another factor, historically mostly Fused Deposition Modeling (FDM) printers were used; however, selective laser sintering (SLS) technology is becoming more and more available, as well as multi-material printing techniques, including polyjet^19^, New methods, and materials are vital as they can lead to new approaches such as bio-inspired ones^20,21^. Also, the advantage of soft robotics has recently been reported in non-expert myo-electric users^22^.

But most important are patients’ needs, and those include functionality desired for the tasks performed in everyday life, which consists of the number of grips that can be performed. This number is dependent on the degrees of freedom, and those range from 4 for Michelangelo^23^ through 6 for i-limb^24,24^ or VICENTevo3^25^ up to even 8 for TASAKA^26,26^. However, this does not translate directly to the number of grips performed can vary from 6 to 24^27^, although the definition of grip position is sometimes treated differently. Thus, even a slight change of the hand position is treated as a separate position. There is a metric system proposed in a study^28^ where the performance is benchmarked by fingertip poses. Recently, haptic feedback methods were reported to help obtain more dexterity with a hand^29^. The low mass of the device is also crucial, as in a survey^30^, many patients found their devices as heavy. Moreover, limited kinematic or motion possibilities and a lack of adjustability to individual size can result in patients’ dissatisfaction and result in a high abandonment rate^31,32^. Therefore, the device must correctly perform the prehension patterns.

To design a prosthetic hand that can do those grips or any other grip, the design process should include the steps to make it possible. This can be done by determining the forces needed to perform a given grip by either experiment or analytically and then building a mechanism that can provide such forces on the fingers. The literature and technical documentation for prosthetics usually describes the forces that a device can achieve. These are in ranges of about 70–140 N^33–36^, but these are general specifications of a device, not for fingers. The calculations of how much force is needed to perform a given grip are less frequent. A study^37^ developed an advanced hand model that evaluated kinematics, but for a pinching grip. Cylindrical grip evaluations were performed experimentally on subjects grabbing smaller objects^38^ and with other masses^39,40^ and often without the measurement of force on the metacarpal area. One study had similar parameters to ours, but it involved no thumb^41^. There were also studies on subjects that were performed on multiple grip sizes, but they focused on maximum obtainable forces^42^(power grips) in cylindrical grips. What was interesting, though, is that with the increasing diameter of the grabbed object, the maximum force decreases^43^. The downside of experiments is usually the cost of sensors and subject involvement.

Some researchers approached the problem with Finite Element Analysis (FEA), which is a powerful tool, especially for non-linear contacts between fingers and objects^44^. This approach allows the designer to investigate any kind of shape or geometry. Still, those methods usually require commercial numerical software, e.g., ANSYS. There is a need for a methodology that wouldn’t require experiments and be universal. For design purposes, analytical methods are beneficial from a cost perspective. Therefore, it was decided to build a model from scratch and analyze it using the proposed method as well as validate it.

**Figure 1:**
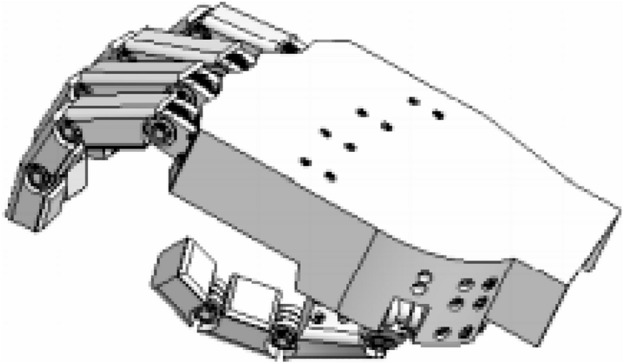
Computer aided design (CAD) model used in testing.

This study aims to develop an analytical model that could be used for estimating forces necessary to perform any variation of grips, especially the hook and cylindrical grip of hand prosthesis. The main advantage could be the reduced cost of an analytical method compared to the cost of the experiment. The idea was to obtain a method that can be used for different sizes of the hand and various designs. The first Computer Aided Design (CAD) model was developed for testing and computational purposes. Its 3D view is visible in Fig. 1.

The following assumptions were imposed on a project:

- Prosthetic size must correspond to the size of a hand
- The Mechanism is to have 18 degrees of freedom

Three degrees for each of the II–V fingers for a total of 12 degrees:

- 1st—for flexion at the distal interphalangeal and proximal interphalangeal joints
- 2nd—allowing flexion in the metacarpophalangeal joint
- 3rd—allowing abduction in the metacarpophalangeal joint

Three degrees of freedom of the thumb.

- 1st—allowing flexion in the interphalangeal joint of the thumb and the metacarpophalangeal joint of the thumb
- 2nd—enabling the adduction and abduction of the thumb
- 3rd—allows you to oppose the thumb

Three degrees of wrist freedom—determining the orientation of the hand in space (the design of the wrist is not included in this work).

- The device must be able to lift 1 kg using a cylindrical grip corresponding to a weight of a 1 L water bottle and 1 kg in hoop grip for easy manipulation of lightweight objects.
- Anthropomorphism and esthetic design
- The mechanism must be enclosed within the prosthesis.
- Ability to perform 14 types of grips and possibility of performing gestures.
- Silent work
- Safety coefficient for the mechanism assumed as n = 1.8.

## Methods and results

### Study design

The first step was to develop a 3D model for calculational purposes. The model would include all the assumptions from the introduction. Next, the analytical mechanics method for calculating forces acting on phalanges in cylindrical grip will be prepared and then solved numerically. The methodology will be based on creating equilibrium equations and determining boundary conditions. To validate the calculations an experiment will be performed on a cylinder with the same dimensions as in the model. To measure the force on phalanges, the force resistive sensors (FSR) will be used similarly to the other experiments found in literature. The results obtained with the method will be later compared to the results obtained in the experiment as well as the reports from the literature. One of the measured outcomes will be the possibility of performing the grip with the forces calculated using proposed methodology and the comparison of load distribution on the fingers.

### Description of model for calculation purposes

The human hand does not have strictly imposed dimensions. Its size, length of phalanges, and their position are individual traits of every human being. This does not limit the ability to grip, manipulate objects or gesticulate in the case of a fully functioning upper limb. That is why in the design process of a prosthesis a few corrections were made to improve the functionality of the prosthetic hand.

**Figure 2:**
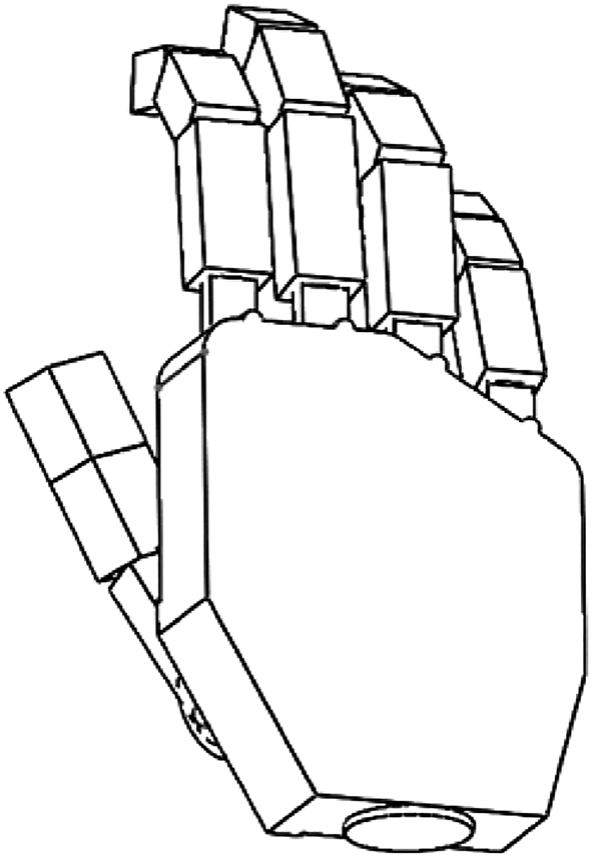
Solid model of the hand created for modeling of the grips.

The simplified model (Fig. 2) consisted of an element of the metacarpal part of the hand, four elements with the possibility of uniaxial rotation that model abduction–adduction of II-V fingers, and twelve phalanges (three for each of the II-V fingers) with the same rotation ability.

For the thumb, folding the first two elements from the proximal side, analogous to the remaining fingers, simulated abduction–adduction as well as opposing the thumb. Further two elements modeling the proximal and distal phalanges of the thumb allowed to model the flexion–extension of the interphalangeal and metacarpophalangeal joints of the thumb.

The hand orientation was not simulated because it was rigidly assigned to the tested variant. The dimensions of the metacarpals and the arrangement of the fingers were taken from ^45^ and ^46^.

**Figure 3:**
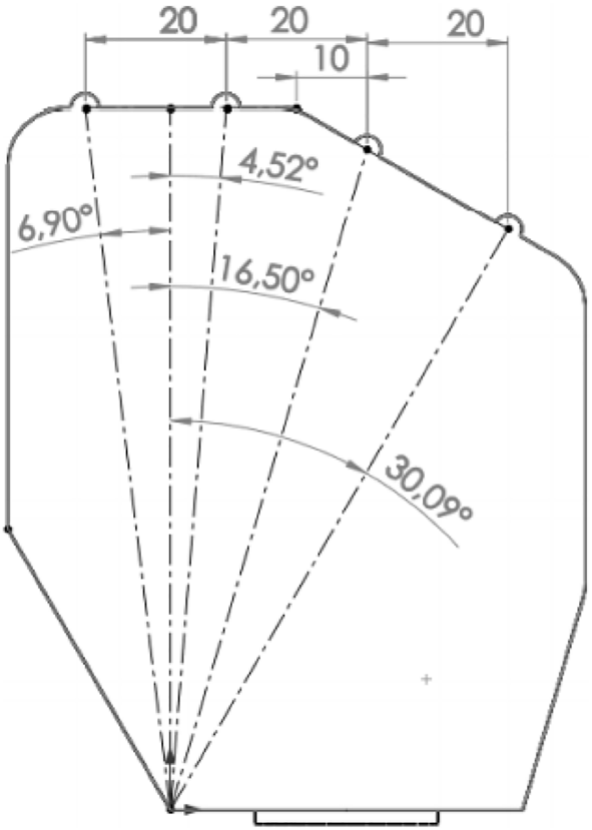
Dimensioning and location of the abduction connections of the metacarpophalangeal joints.

**Figure 4:**
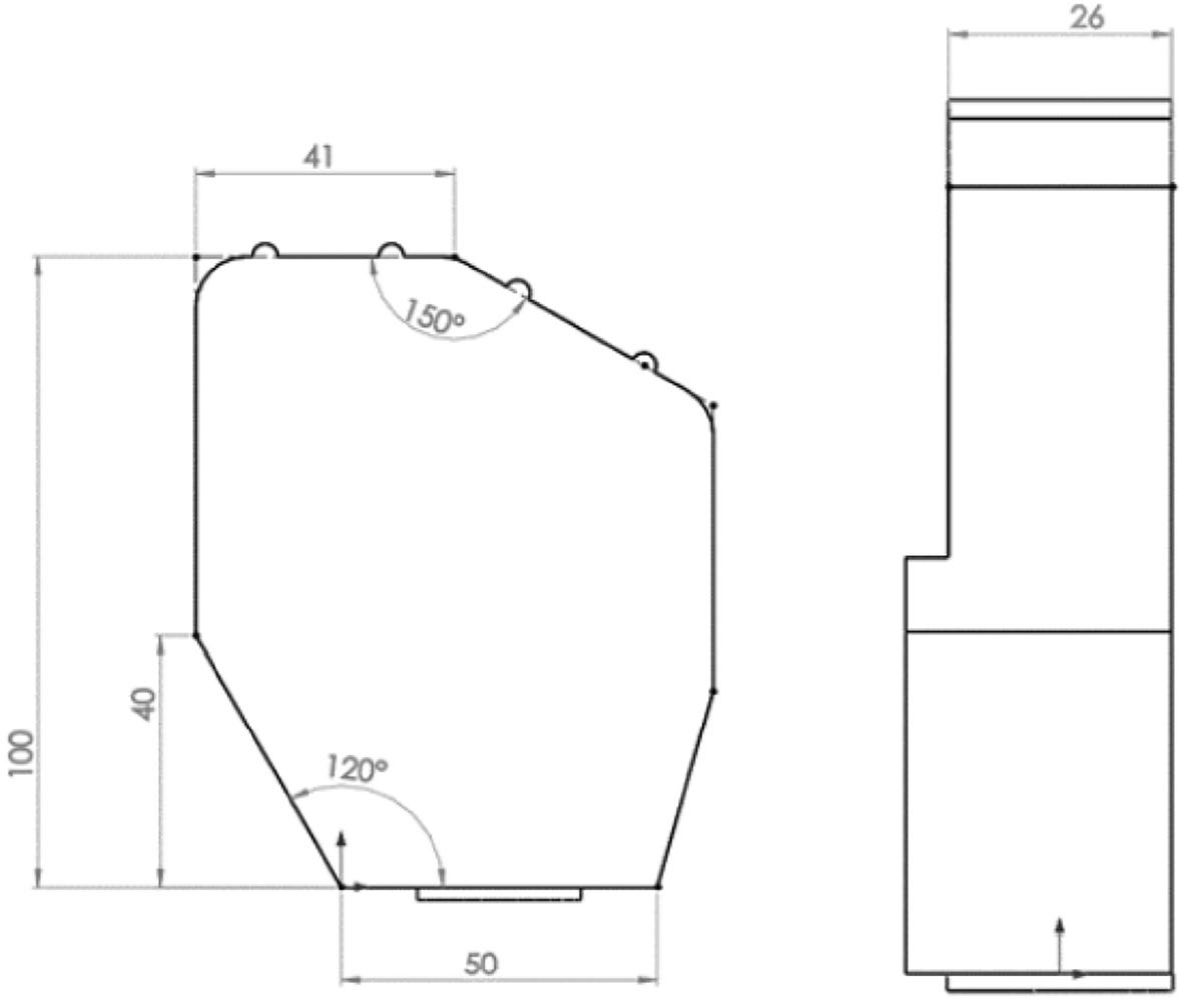
Dimensions of the metacarpal element of the hand model used for further calculations.

Minor changes have been made to facilitate the design of the prosthesis mechanism, e.g. to reduce the variety of mechanisms and maximize the use of a small design space, the distances between individual phalanges were unified. The metacarpal part has been modeled as one rigid body (Fig. 3) in the shape of a prism with an additional structure that allows to reproduce the movement of the thumb in relation to the inner surface of the hand. The surface containing the axis of rotation of the II-III fingers in the abduction–adduction movement was located at a distance of 100 mm in relation to the selected coordinate system, and the axis of rotation of the other fingers was placed on the surface situated at an angle of 150 degrees and with an offset of 41 mm (Fig. 4). The model thickness for most of the metacarpal part was assumed to be 26 mm, approximately the same as in^46^.

**Figure 5:**
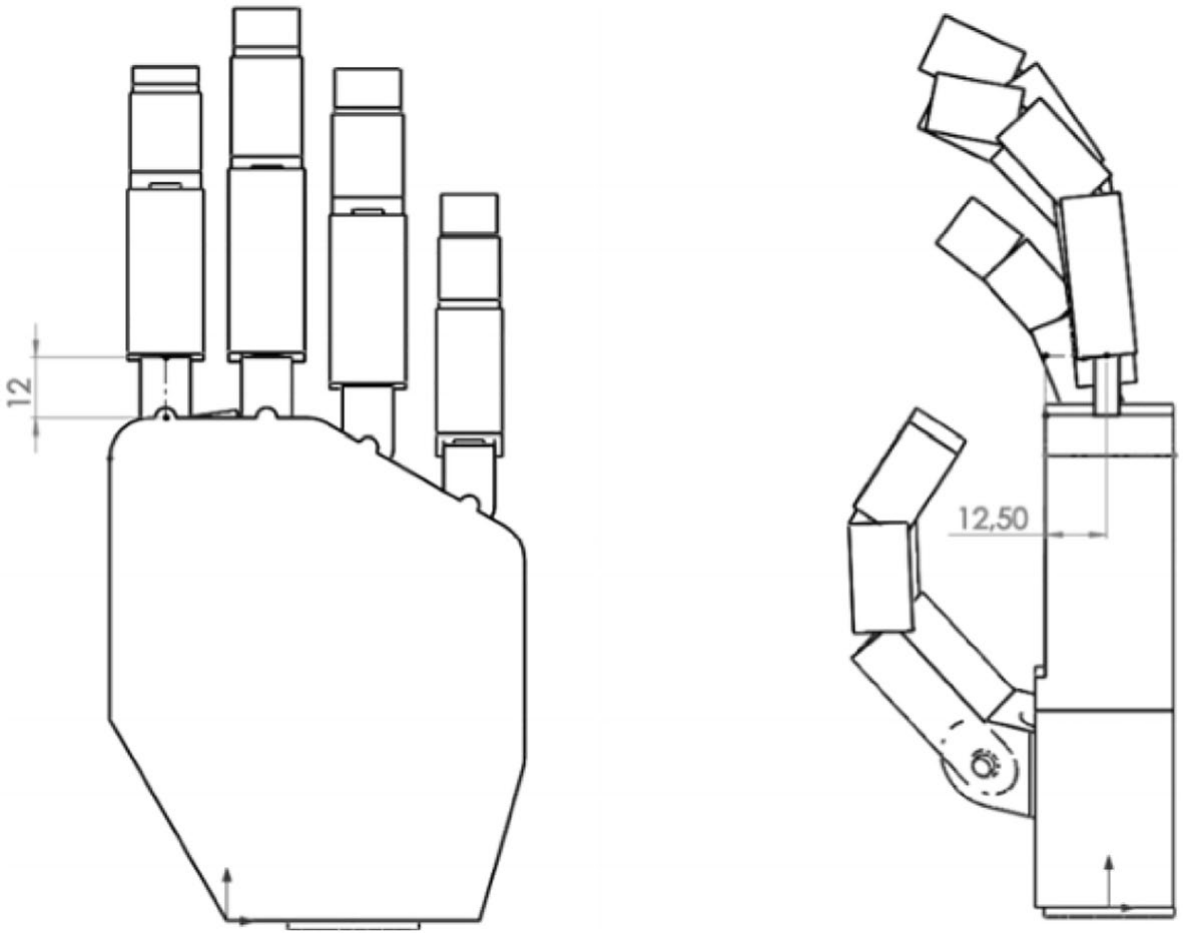
Important dimensions of the modeled element for the metacarpophalangeal joint i.e. the position of the bending axis in the metacarpophalangeal joint of the II-V fingers in the solid model.

The axes of rotation were modeled as cylindrical surfaces placed in fixed positions. The axis of rotation for bending was shifted by 12 mm, which enabled to simulate the length of the designed abduction mechanism It was positioned at a distance of 12.5 mm from the inner surface of the metacarpal part, which also corresponds to the mechanical model of the prosthesis. The bending rotation axes were modeled as cylinders with the axis passing through the centers of the frontal phalanges. The whole assembly was mounted on an element enabling the abduction–adduction movement and attached to the metacarpal part (Fig. 5).

The fingers were modeled with the sizes listed in (Table 1)

**Table 1:** Segment lengths and widths of the modeled II-V fingers.

Finger	Length of phalanx [mm]			Width of phalanx [mm]		
	DIP	MIP	PIP	DIP	MIP	PIP
Pointing	18	21.3	33	15	16	17
Middle	18	23.9	37	15	16	17
Ring	18	23.1	33.6	15	16	17
Little	15	16.3	26.8	13	14	15

DIP—distal phalanx, MIP—middle phalanx, PIP—proximal phalanx.

The surface of the rotation axis location for the thumb was tilted 120 degrees from the vertical. This was done to obtain a high functionality of the hand, but it omits the full anthropomorphism.

**Figure 6:**
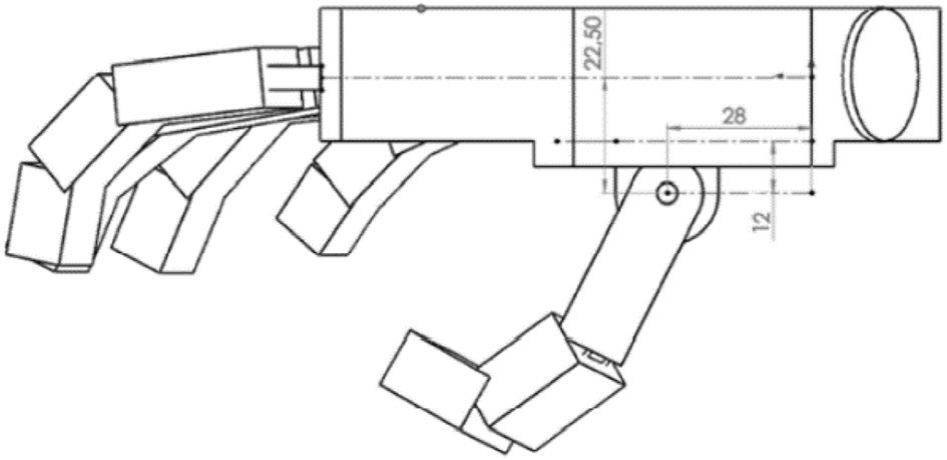
The position of the thumb in relation to the metacarpal part and the abduction–adduction axis of the thumb.

**Figure 7:**
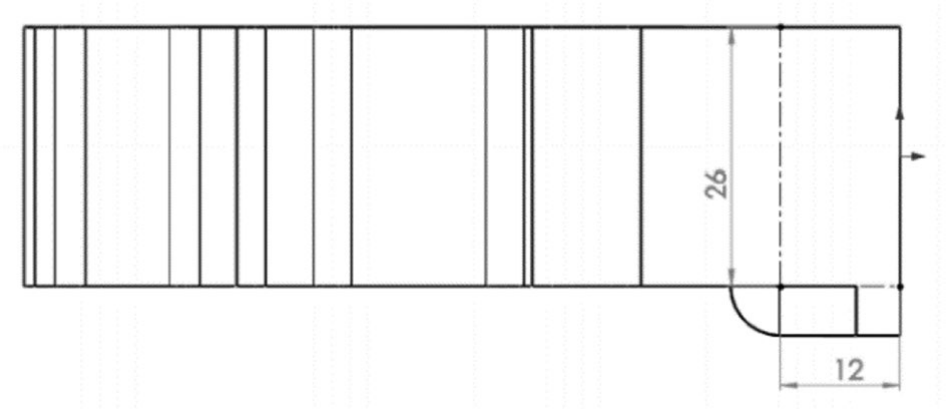
Position of the thumb opposing axis relative to the plane of the thumb and the top of the hand location.

The first axis of rotation is located 12 mm from the above-mentioned surface and 22.5 mm (Fig. 6) below the plane containing the axes of rotation, simulating bending in the metacarpophalangeal joints of II–V fingers. The second axis (Fig. 7) was moved away by 28 mm from the adopted coordinate system along the wall, on which the thumb was placed, and by 12 mm from the previous axis of rotation, leaving room for the mechanism to be placed in this space in the mechanical model.

**Figure 8:**
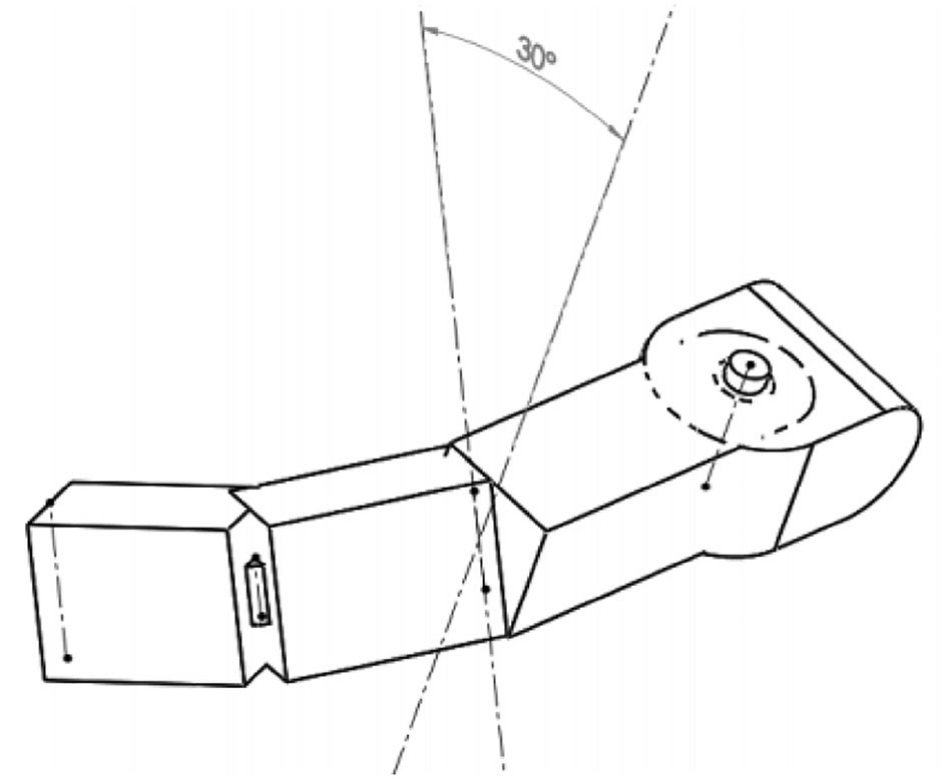
Characteristic rotation of the metacarpophalangeal joint of the thumb used in the model.

A characteristic feature of the modeled thumb was the rotation of the metacarpophalangeal joint of the thumb by 30 degrees i.e., tilting the lower part of the finger towards the inner part of the hand (Fig. 8). This procedure allowed for the efficient grasping of oval objects and significantly helped in mapping the opposition of the human thumb. Its dimensions were as presented in Table 2**.**

**Table 2:** Thumb segments lengths and widths.

	DIP	PIP	ABD
Length [mm]	18.3	25.9	34
Width [mm]	20	20	14

DIP-distal phalanx, PIP proximal phalanx, ABD-opposing element.

The length of the thumb metacarpal was selected experimentally, maximizing the anthropomorphism of the grip and minimizing the forces exerted on the tested model.

**Figure 9:**
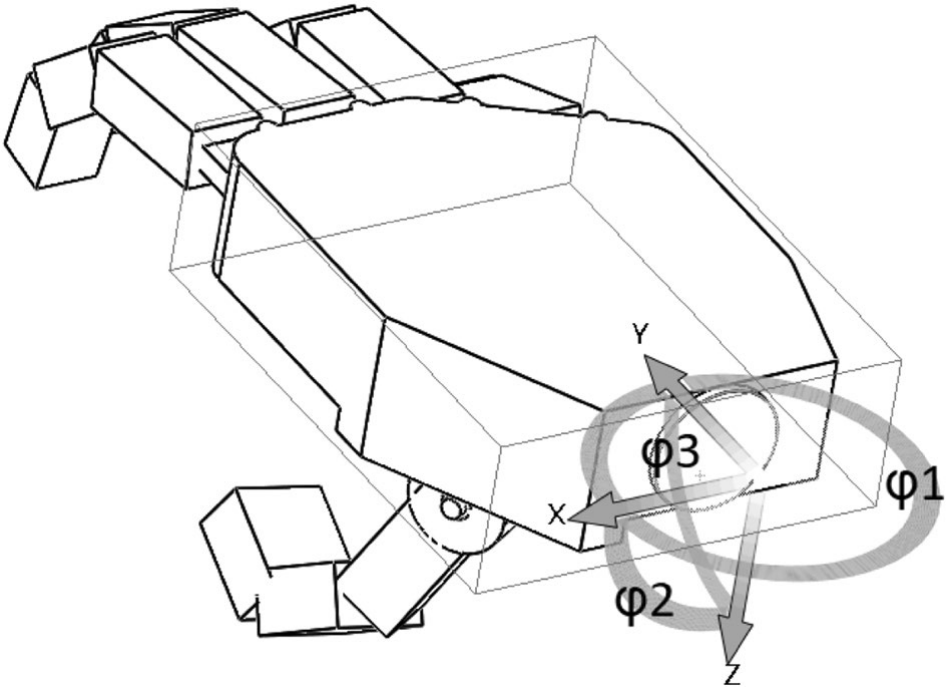
Control angles in the wrist that define the orientation of the hand.

The height of each of the phalanges was set at 13.5 mm. Due to the shift of the axis of rotation in the mechanical model of the prosthesis by 0.75 mm below the center of the phalanx (which is 6 mm from its lower wall), the height of the phalanges was assumed as 12 mm in the calculation model, which allowed for easier modeling of the fingers’ segments. Thanks to this procedure, the correctness of the obtained results was ensured. The height of the segment modeling the metacarpal bone of the thumb was modeled as equal to its width (14 mm). The designed mechanism was considered to be five open kinematic chains, one for each finger and thumb, attached to the casing of the metacarpal part of the hand (Fig. 9).

The kinematic of the index (i = 2), middle (i = 3), ring (i = 4) and little (i = 4) finger is described by four angles:

- ϴ_*i*1_—corresponding to the flexion in the distal interphalangeal joint,
- ϴ_*i*2_—responsible for flexion in the proximal interphalangeal joint,
- ϴ_*i*3_—responsible for the flexion in the metacarpophalangeal joint,
- ϴ_*i*4_—responsible for abduction in the metacarpophalangeal joint.

Additionally, four angles are defined for the thumb (i = 1):

- ϴ_*i*1_—the bending angle between the distal and proximal phalanges of the thumb,
- ϴ_*i*2_—the angle of flexion in the metacarpophalangeal joint of the thumb,
- ϴ_*i*3_—angle responsible for the adduction and abduction of the thumb,
- ϴ_*i*4_—the angle responsible for opposing the thumb.

The ranges of all angles are given in Table 3, where 0˚ means a complete extension of the finger in relation to the metacarpal plane or maximum extension of the II-V fingers in the y direction. The angle ϴ_14_ = 0° means the position of the thumb parallel to the metacarpal surface of the hand, and the angle ϴ_13_ = 0° means the position of the thumb is perpendicular to the surface of its fixture.

**Table 3:** Ranges of angle motion between each of the phalanges in the created model.

Finger	*i*	ϴ_*i*1_, ϴ_*i*2_, ϴ_*i*3_ (deg)	ϴ_*i*4_ (deg)
Thumb	1	0–90	0–150
Pointing	2	0–90	(−20)–20
Middle	3	0–90	(−20)–20
Ring	4	0–90	(−20)–20
Little	5	0–90	(−20)–20

### Cylindrical grip

**Figure 10:**
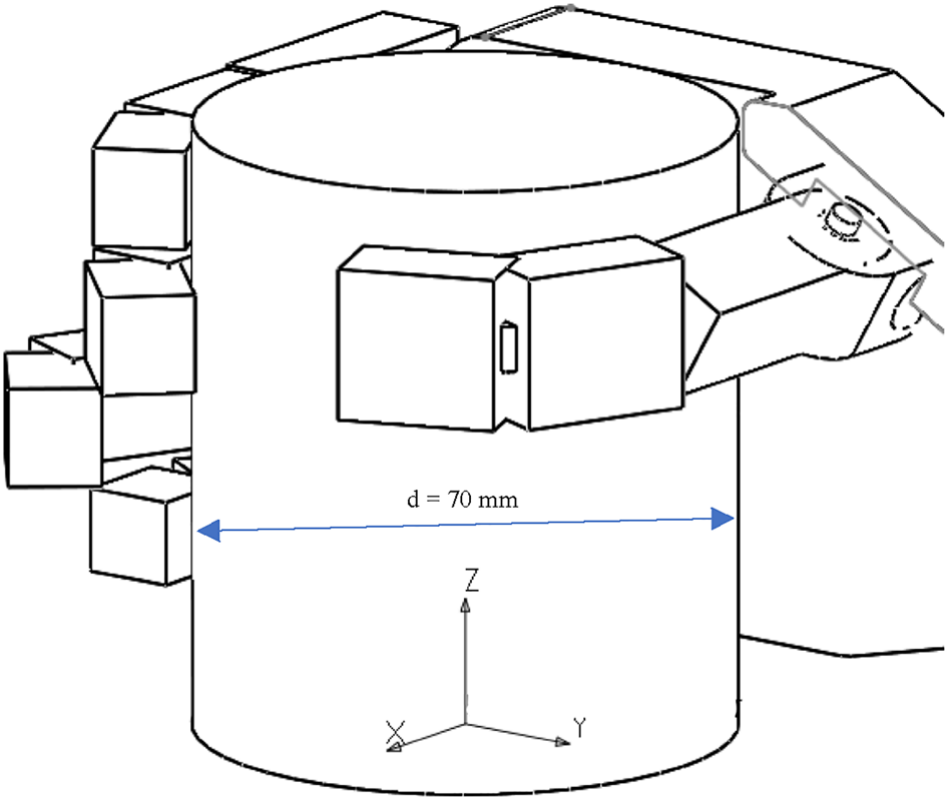
The cylindrical grip model. Setting of the fingers on the tested cylinder with assumed coordinate system (XYZ) for static calculations.

The mechanism described previously will be used to test the method for calculating the forces in the cylindrical grip, which imitates everyday activities such as holding a water bottle and the second can be used for carrying objects. The model (Fig. 10) was created for a cylindrical grip on a cylindrical block with a diameter d = 70 mm and a height H = 82 mm equal to the width of the tested hand. This diameter was selected by measuring several popular, generally available water bottles. Its positioning in space depends on the contact with all the phalanges belonging to the fingers that hold the cylinder’s weight. In this model, we assumed that only the thumb, index, and middle finger are in contact with the cylinder. The assumption was made based on the results of preliminary calculations with the participation of all fingers. The results showed that the forces for the ring and little finger are significantly lower than for the other fingers. Also, many hand prosthesis devices use only 3 fingers due the limited number of driving motors. Therefore, by omitting them in the calculations, no major error was made. Moreover, the obtained results, due to their overestimation, resulted in the increased reliability of the model's strength an additional condition was the parallel arrangement of all phalanges, which made the calculations much easier.

**Figure 11:**
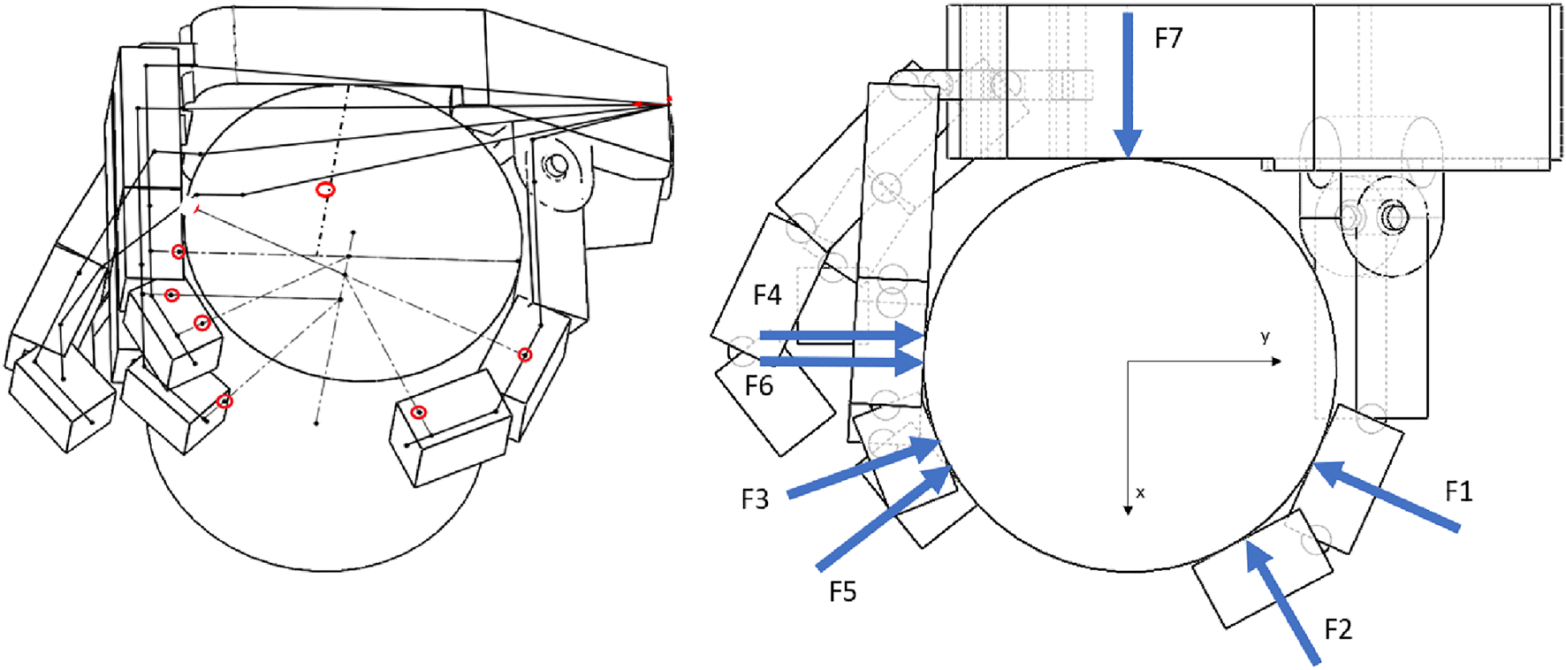
Model in cylindrical grip with a sketch to read the points of contact (in red) between the model and the cylinder in the assumed reference system (left) and a top view with forces acting on a cylinder (right). Forces: F1,F2—Thumb; F3,F4—Index; F5,F6—Middle and F7 Metacarpal.

A 3D sketch (Fig. 11) was made for the selected position of a model. Contact points between block and phalanges and directions of forces were obtained by creating lines perpendicular to the axis of cylinder and the line that connects midpoints of the frontal and aft walls of the phalanges.

In order to estimate the loads on the model the number of unknowns had to be limited. It was assumed that each of the fingers exerts the same force on the cylinder at each point of contact between the phalanx and the cylinder. The model revealed that for the index finger, contact occurs for two distal and middle phalanges, the same situation excluding the involvement of the proximal phalanx occurred for the middle finger. Two points of contact were also read for the thumb on both of its segments. In the middle of the contact length of the inner part of the metacarpus, the concentrated force transmitted by this segment was assumed. A total of seven touchpoints were obtained. A system of statics equations was developed for the block with four unknowns: the force acting from the thumb, the force acting from the index finger, the force acting from the middle finger, and the force coming from the metacarpal part. The first three of which act from the two phalanges of the corresponding finger, differing in the point of application and direction. It was assumed that the forces selected in this way are to transfer the weight of the modeled water bottle. Here the own weight of the phalanges is not considered as it is analytical model used for calculating forces on fingers in a grip. However, the mechatronics model of the device should contain such calculation and the transferring forces are not negligible.

Next the equilibrium Eqs. (1–6) for the block were written in the adopted coordinate system located at the point of intersection of the cylinder base and its axis of symmetry as shown in the above Fig. 11. System consists of 3 equations for equilibrium of forces (1–3) in three directions of space (x,y,z) and three equations for equilibrium of moments (4–6) as below:

1 $$\sum _{i=1}^{n}F{x}_{i}=0$$

2 $$\sum _{i=1}^{n}F{y}_{i}=0$$

3 $$\sum _{i=1}^{n}F{z}_{i}=m\cdot g$$

4 $$\sum _{i=1}^{n}(F{y}_{i}\cdot {z}_{i}-F{z}_{i}\cdot {y}_{i})=0$$

5 $$\sum _{i=1}^{n}(F{z}_{i}\cdot {x}_{i}-F{x}_{i}\cdot {z}_{i})=0$$

6 $$\sum _{i=1}^{n}(F{x}_{i}\cdot {y}_{i}-F{y}_{i}\cdot {x}_{i})=0$$

where:

g is the gravitational acceleration, here assumed as 9.81 $m\cdot {s}^{-2}$;

m is the mass of the cylinder/bottle, in this case 1000 g;

n is number on points of contact between the prosthesis and the cylinder, in this case n = 7 since there are 7 contact points;

Fx_i_, Fy_i_, Fz_i_ are forces in the x,y and z direction respectively on each of the contact points. The force Fz was the result of the friction by which the block was held;

The coordinates (*x*_*i*_, *y*_*i*_, *z*_*i*_) are points of force application. They were read from the 3D model.

As a result of the number of contact points, there are 21 unknowns in total, which are components of 7 forces in x,y and z direction. Since the number of equations is 6, some assumptions had to be made in order to reduce the number of unknowns to be able to solve the equations. The first was made by assuming that the forces F1 and F2 are the forces of the thumb equal to the module F_TH_ , the forces F3 and F4 are the forces of the index finger equal to the module F_ID_. The forces F5 and F6 analogically correspond to the force of the middle finger F_MD_ , and force F7 = F_MC_ was assigned to the metacarpal part. For visual representation see Fig. 11. Additionally, taking into account the fact that each force acts horizontally on the cylinder, vertical force components will be equal to 0. Using those assumptions, the above formulas of equilibriums (1–6) can be rewritten as follows (7–12):

7 $${F}_{TH}\sum _{i=1}^{2}cos\left({\alpha }_{i}\right)+{F}_{ID}\sum _{i=3}^{4}cos\left({\alpha }_{i}\right)+{F}_{MD}\sum _{i=5}^{6}cos\left({\alpha }_{i}\right)+{F}_{MC}cos\left({\alpha }_{7}\right)=0$$

8 $${F}_{TH}\sum _{i=1}^{2}sin\left({\alpha }_{i}\right)+{F}_{ID}\sum _{i=3}^{4}sin\left({\alpha }_{i}\right)+{F}_{MD}\sum _{i=5}^{6}sin\left({\alpha }_{i}\right)+{F}_{MC}sin\left({\alpha }_{7}\right)=0$$

9 $${F}_{TH}\sum _{i=1}^{2}\mu +{F}_{ID}\sum _{i=3}^{4}\mu +{F}_{MD}\sum _{i=5}^{6}\mu +{F}_{MC}\cdot \mu =m\cdot g$$

10 $$ \begin{aligned}  & \sum _{i=1}^{2}({F}_{TH}\cdot s\mathrm{in}\left({\mathrm{\alpha }}_{i}\right)\cdot {z}_{i}-{\mathrm{F}}_{TH}\cdot\upmu \cdot {y}_{i})+\sum _{i=3}^{4}({F}_{ID}\cdot s\mathrm{in}\left({\mathrm{\alpha }}_{i}\right)\cdot {z}_{i}-{F}_{ID}\cdot\upmu \cdot y\mathrm{i}) \\&\quad  +\sum _{i=5}^{6}({F}_{MD}\cdot s\mathrm{in}\left({\mathrm{\alpha }}_{i}\right)\cdot {z}_{i}-{\mathrm{F}}_{MD}\cdot\upmu \cdot y\mathrm{i})+{F}_{MC}\cdot s\mathrm{in}\left(\mathrm{\alpha }7\right)\cdot {z}_{7}-{F}_{MC}\cdot\upmu \cdot {y}_{7}=0\end{aligned} $$

11 $$ \begin{aligned}  & \sum _{i=1}^{2}({F}_{TH}\cdot \mu \cdot {x}_{i}-{F}_{TH}\cdot cos\left({\alpha }_{i}\right)\cdot {z}_{i})+\sum _{i=3}^{4}({F}_{ID}\cdot \mu \cdot {x}_{i}-{F}_{ID}\cdot cos\left({\alpha }_{i}\right)\cdot {z}_{i})  \\ & \quad +\sum _{i=5}^{6}({F}_{MD}\cdot \mu \cdot {x}_{i}-{F}_{MD}\cdot cos\left({\alpha }_{i}\right)\cdot {z}_{i})+{F}_{MC}\cdot \mu \cdot {x}_{7}-{F}_{MC}\cdot cos\left({\alpha }_{7}\right)\cdot {z}_{7}=0\end{aligned} $$

12 $$ \begin{aligned}  & \sum _{i=1}^{2}({F}_{TH}\cdot cos\left({\alpha }_{i}\right)\cdot {y}_{i}-{F}_{TH}\cdot sin\left({\alpha }_{i}\right)\cdot {x}_{i})+\sum _{i=3}^{4}({F}_{ID}\cdot cos\left({\alpha }_{i}\right)\cdot {y}_{i}-{F}_{ID}\cdot sin\left({\alpha }_{i}\right)\cdot {x}_{i})\\ &\quad+\sum _{i=5}^{6}({F}_{MD}\cdot cos\left({\alpha }_{i}\right)\cdot y-{F}_{MD}\cdot sin\left({\alpha }_{i}\right)\cdot {x}_{i})+{F}_{MC}\cdot cos\left({\alpha }_{7}\right)\cdot {y}_{7}-{F}_{MC}\cdot sin\left({\alpha }_{7}\right)\cdot {x}_{7}=0\end{aligned} $$

where:

the friction coefficient was assumed as for the contact between plastic and rubber—µ = 0.6

${\alpha }_{i}$ is an angle that defines the direction of the forces applied to the phalanges, detailed explanation below;

F_INDEX_ is force acting between phalange and cylinder described in the paragraph above.

Thanks to this, the number of unknowns was reduced to 4, which is less than the number of equations available and means that the solution can be found. To solve the equation, it was also necessary to determine the angle (α) defining the direction of the forces applied to the phalanges. For this purpose, the 2-argument arctangent was used (Eq. 13). It is a function used in programming languages to find an angle between the ray from the origin to the point (x,y). Or alternatively a phase angle of a complex number.

13 $${\alpha }_{i}=atan2 \left({y}_{i},{x}_{i}\right)-\pi ;$$

The π shift was made to adjust the sense/direction of the forces acting on the cylinder.

Finally, the equations were written in the matrix form M ∙ F = Q.

where:

M—system dependence matrix (Eq. 14).

F—matrix of the forces sought (Eq. 15).

Q—system load matrix (Eq. 16).

14 $$\mathrm{M}=\left[\begin{array}{cccc}cos\left({\alpha }_{1}\right)+cos\left({\alpha }_{2}\right)& cos\left({\alpha }_{3}\right)+cos\left({\alpha }_{4}\right)& cos\left({\alpha }_{5}\right)+cos\left({\alpha }_{6}\right)& cos\left({\alpha }_{7}\right)\\ sin\left({\alpha }_{1}\right)+sin\left({\alpha }_{2}\right)& sin\left({\alpha }_{3}\right)+sin\left({\alpha }_{4}\right)& sin\left({\alpha }_{5}\right)+sin\left({\alpha }_{6}\right)& sin\left({\alpha }_{7}\right)\\ 2\mu & 2\mu & 2\mu & 1\mu \\ {X}_{TH}& {X}_{ID}& {X}_{MD}& {X}_{MC}\\ {Y}_{TH}& {Y}_{ID}& {Y}_{MD}& {Y}_{MC}\\ {Z}_{TH}& {Z}_{ID}& {Z}_{MD}& {Z}_{MC}\end{array}\right]$$

15 $$F=\left[\begin{array}{c}{F}_{TH}\\ {F}_{ID}\\ {F}_{MD}\\ {F}_{MC}\end{array}\right]$$

16 $$Q=\left[\begin{array}{c}0\\ 0\\ m\cdot g\\ 0\\ 0\\ 0\end{array}\right]$$

For parameters X,Y,Z given in Eqs. 17–28:

17 $${X}_{TH}=\sum_{i=1}^{2}(\mathrm{sin}\left({\alpha }_{i}\right)\cdot {z}_{i} -\mu \cdot {y}_{i})$$

18 $${X}_{ID}=\sum_{i=3}^{4}(\mathrm{sin}\left({\alpha }_{i}\right)\cdot {z}_{i} -\mu \cdot {y}_{i})$$

19 $${X}_{MD}=\sum_{i=5}^{6}(\mathrm{sin}\left({\alpha }_{i}\right)\cdot {z}_{i} -\mu \cdot {y}_{i})$$

20 $${X}_{MC}=(\mathrm{sin}\left({\alpha }_{7}\right)\cdot {z}_{7} - \mu \cdot {y}_{7})$$

21 $${Y}_{TH}= \sum_{i=1}^{2}(\mu \cdot {x}_{i} -\mathrm{cos}\left({\alpha }_{i}\right)\cdot {z}_{i)}$$

22 $${Y}_{ID}=\sum_{i=3}^{4}(\mu \cdot {x}_{i} -\mathrm{cos}\left({\alpha }_{i}\right)\cdot {z}_{i})$$

23 $${Y}_{MD}=\sum_{i=5}^{6}(\mu \cdot {x}_{i} -\mathrm{cos}\left({\alpha }_{i}\right)\cdot {z}_{i})$$

24 $${Y}_{MC}=(\mu \cdot {x}_{7} -\mathrm{ cos}\left({\alpha }_{7}\right)\cdot {z}_{7})$$

25 $${Z}_{TH}=\sum_{i=1}^{2}(\mathrm{cos}\left({\alpha }_{i}\right)\cdot {y}_{i} -\mathrm{sin}\left({\alpha }_{i}\right)\cdot {x}_{i})$$

26 $${Z}_{ID}=\sum_{i=3}^{4}(\mathrm{cos}\left({\alpha }_{i}\right)\cdot {y}_{i} -\mathrm{sin}\left({\alpha }_{i}\right)\cdot {x}_{i})$$

27 $${Z}_{MD}=\sum_{i=5}^{6}(\mathrm{cos}\left({\alpha }_{i}\right)\cdot {y}_{i} -\mathrm{sin}\left({\alpha }_{i}\right)\cdot {x}_{i})$$

28 $${Z}_{MC}=(\mathrm{cos}\left({\alpha }_{7}\right)\cdot {y}_{7} - \mathrm{sin}\left({\alpha }_{7}\right)\cdot {x}_{7})$$

**Figure 12:**
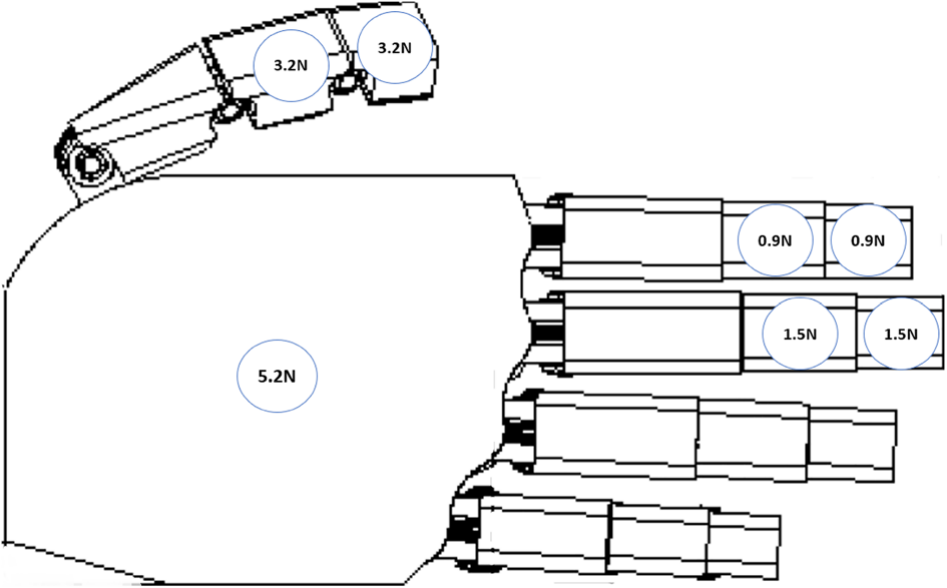
Modulus of forces acting on the load-transferring phalanges and the metacarpal part in the cylindrical grip (1 kg, d = 70 mm) calculated using the analytical method.

The problem was solved by implementing the appropriate code in the MATLAB software using the equation: F = M\Q and the resulting modules of forces acting on phalanges and the metacarpal part were obtained (F_TH_, F_ID_, F_MD_ and F_MC_). The values are given in the Fig. 12, it should be noted that value predicted for metacarpal is higher, but the number of contacts there was limited to one.

To verify whether the results match the actual forces in the investigated grip, experiments had to be performed as there was no report of the exact geometry and load in literature. The goal was to confirm the distribution of forces, the total sum of forces and the possibility of performing the grip in the way it was modeled. The following experiment was performed by the author of the manuscript on own hand, without the involvement of any patients.

### Validation of the obtained forces

**Figure 13:**
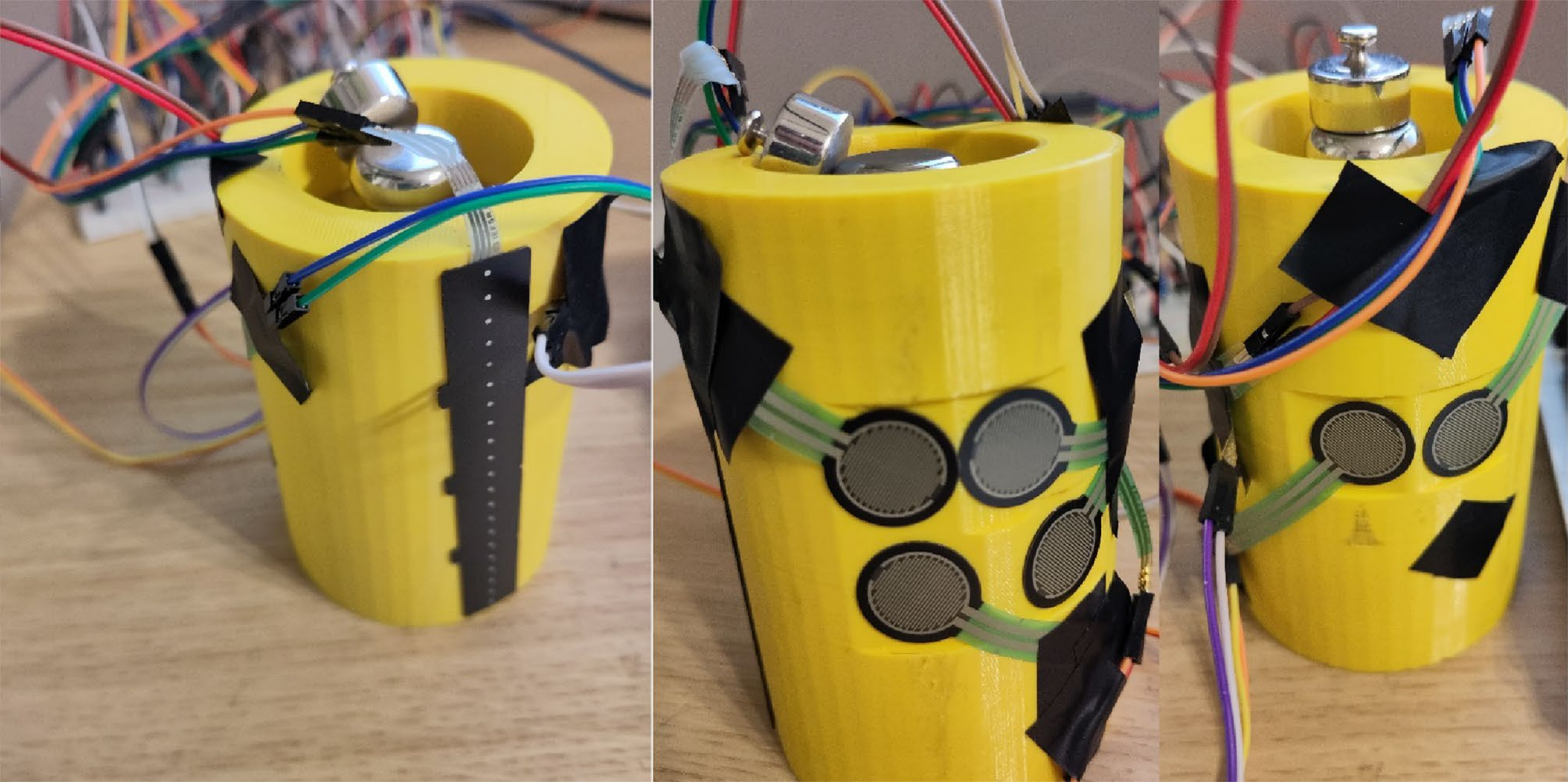
Setup for the cylindrical grip: left—FSR strip for reading the pressure on the metacarpal part; middle—FSR sensors for index and middle finger phalanges; right—FSR sensors for thumb phalanges.

It was decided to use the Force Sensitive Resistors (FSR) for measurements as they were available, and the range of forces expected in the experiment matched their measurement range. The downside is that they require calibration and offer mediocre repeatability of measurement cycle-to-cycle. Nevertheless, such a solution was used for the cylindrical grip. The 70 mm cylinder used in the analytical model has been 3D printed with space inside for 500 g and 200 g weights (300 g print) to achieve the 1 kg mass (Fig. 13). A small change in outside geometry was made, as under each contact point with a phalange, the surface has been flattened to obtain a 20 × 20 mm square area to which the FSR sensors can be attached, as it is recommended to perform the measurement on a flat surface.

**Figure 14:**
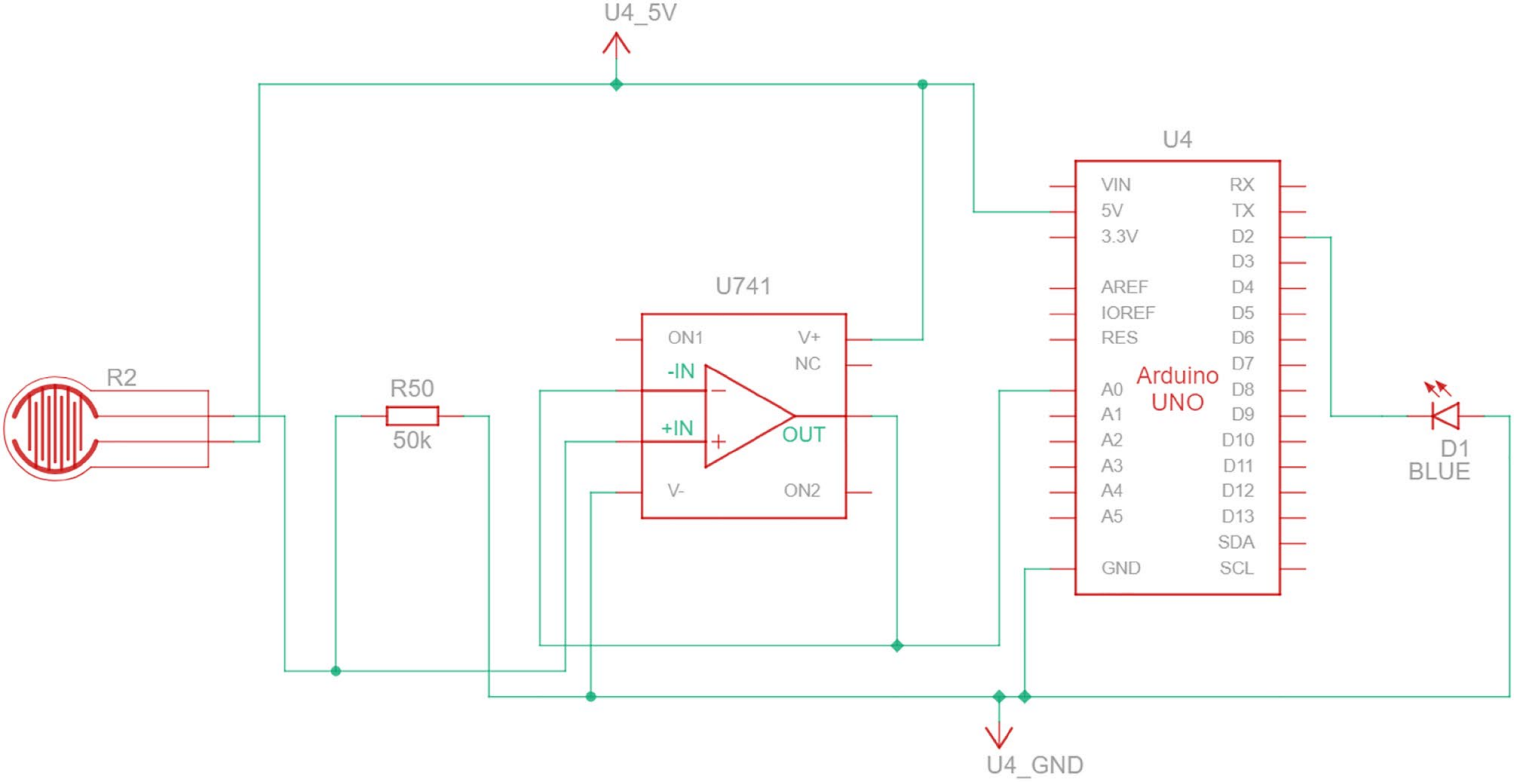
Measurement electronic setup for FSR sensor under finger (repeated for each of the six sensors).

**Figure 15:**
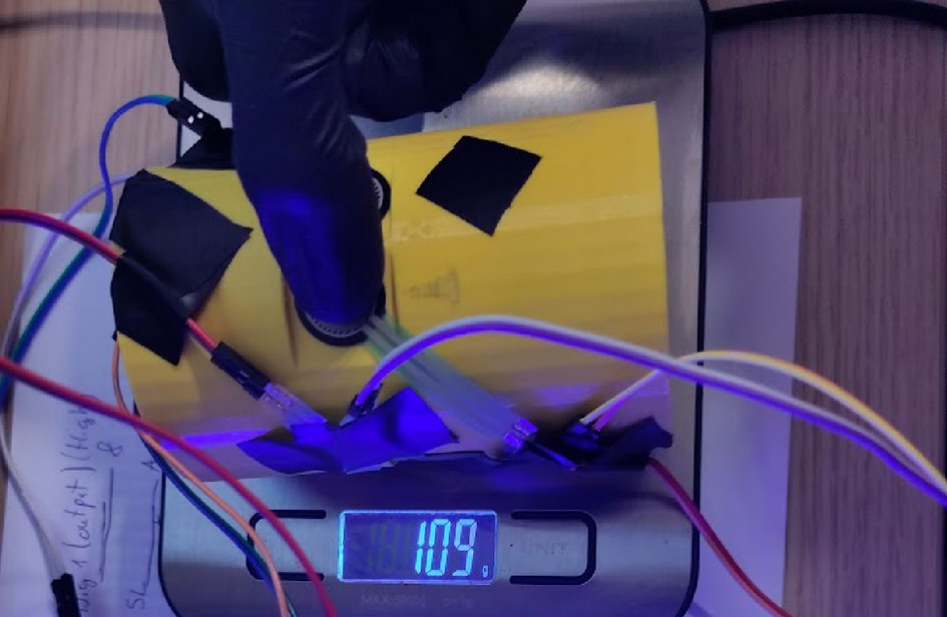
Setup for calibration of FSR sensors in cylindrical grip.

The FSR 402 sensors^47^ were used to gather data from the finger phalanges (six in total). They have 20 mm diameter large measurement area to which the pressure can be applied. The downside of such sensors is that the repeatability of force measurements is far from ideal (+ /- 25%), and the sensors must be calibrated, yet even then the repeatability is around 5–10%. Other sensor had to be used for the metacarpal part of the hand, since the contact patch on a real hand consisted of multiple points along a metacarpal joint line. In the experiment it was decided to place the sensor under the heads of metacarpal bones and perform the grip in such a way that the contact with cylinder occurs only through the sensor. The 2730 sensor^48^ was used, which is a 20 mm wide strip sensor. Other recommendations on how to improve the uncertainty and repeatability made by the manufacturer are stored in the integration guide^49^. It wasn’t possible to follow all of them due to the experiment setup, but the following was done:

- The current-to-voltage circuit was used instead of the voltage divider. This allowed to measure the force more precisely, yet calibration and curve fitting was necessary. For the hardware part, the operational amplifier LM-741 was used with 50 KOhm R0 resistors. All electronics were placed on a breadboard, and the power and data gathering were performed on the Arduino prototyping board. Setup is depicted schematically in Fig. 14.
- Calibration was performed using a digital scale with the cylinder placed on it sideways, with a sensor targeted for calibration on the upper side of the cylinder (Fig. 15). The force was applied directly from above onto a sensor that was supposed to be calibrated. Since the force acted in the same direction as the gravity, i.e., perpendicular to the scale surface, it can be assumed that the force applied to the sensor is equal to the weight read on the scale. The output signal from FSR was matched with a reading on the scale.

This provided the relation between weight measured on the scale (and thus a force) and reference output voltage that could be used later for fitting the data. It was mostly repeatable for the 402 sensors with the same R0 resistor, but different for the metacarpal strip, as it was a 20kOhm. The metacarpal sensor also used the LM324 op-amp and circuit from this open-source project^50^.

- For the initial calibration of the sensors and the final measurement, the same applicators were used (fingers) instead of dead weights, which means the contact patch with a sensor stays similar. The values were read every + /- 100 g and the corresponding FSR reading was stored in the spreadsheet. As mentioned before the resistance was fine-tuned to obtain linear output-to-weight relation between 200 and 400 g (2-4N). This was necessary to limit the uncertainty of the obtained results.
- The friction coefficient used in the model was assumed to be equal to 0.6 for plastic-to-rubber, as those were the mating surface’s materials. This should be reproduced as faithfully as possible; therefore, a nitrile glove was worn during the validation to simulate the contact. The real coefficient might depend on many other factors such as surface roughness, thus it might not be the same for the experiment in the real world. Also, a barehand grip was performed to see how much it influences the result.

**Figure 16:**
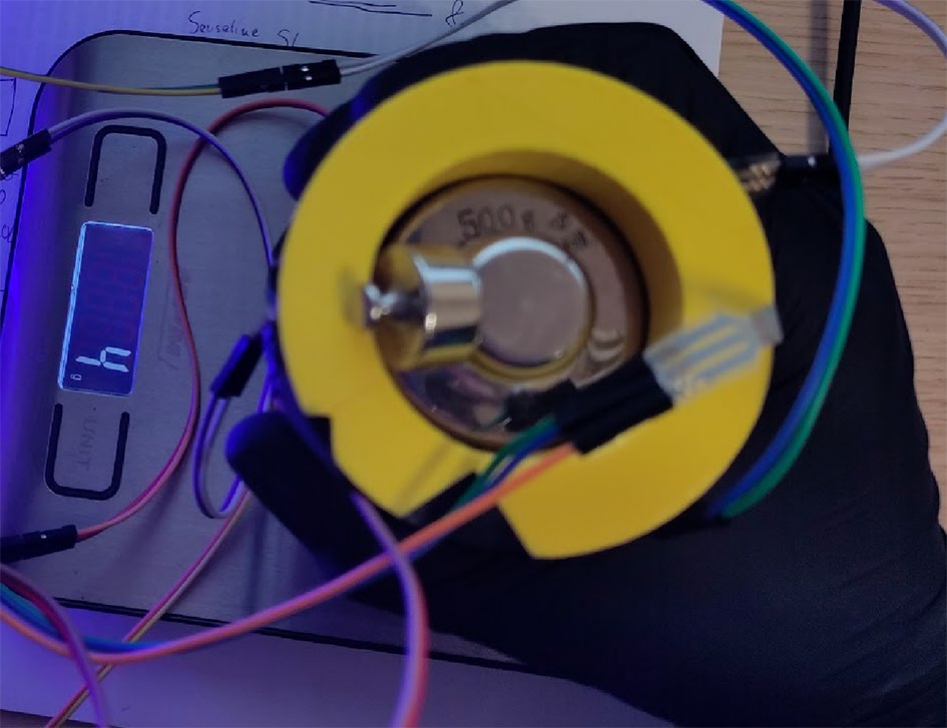
Measurement setup for determining forces acting on phalanges in a grip on the cylinder (d = 70 mm, m = 1 kg) with hand in a nitrile glove.

The measurement procedure was to grab the cylinder by placing each finger on the sensor and aligning the metacarpal part on the strip sensor. A care was taken not to touch the cylinder in places other than the sensor area. Then the cylinder was lifted until the measurement of the weight was equal to zero (Fig. 16). The output was recorded after about 5 s when the reading has stabilized. The obvious bias in the experiment was that the operator could see the values read by the FSRs and knew what the target was. However, this experiment is used to verify if the obtained forces are enough to carry the object and check their distribution.

The gathered data was processed. First, the calibration curves were obtained using points gathered during calibration. Next, the gathered outputs from the measurements were recalculated into the weight on the finger using the linear interpolation (Eq. 29). This was a better way as the relation was linear in the range where forces were expected and measured. Furthermore, low-order polynomials weren’t matching the calibration points and the higher order caused numerical problems.

29 $$x= {y}_{1}+\left(y-{x}_{2}\right)\left(\frac{{y}_{2}-{y}_{1}}{{x}_{2}-{x}_{1}}\right)$$

where:

$y$—The FSR value from measurement that corresponds to unknown weight x.

${y}_{1}$—Closest lower calibration coordinate y output value, read from FSR for a given calibration weigth.

${y}_{2}$—Closest upper calibration coordinate y output value, read from FSR for a given calibration weight.

$$x$$—Unknown weight.

${x}_{1}$—Closest lower calibration coordinate weight value.

${x}_{2}$—Closest upper calibration coordinate weight value.

**Figure 17:**
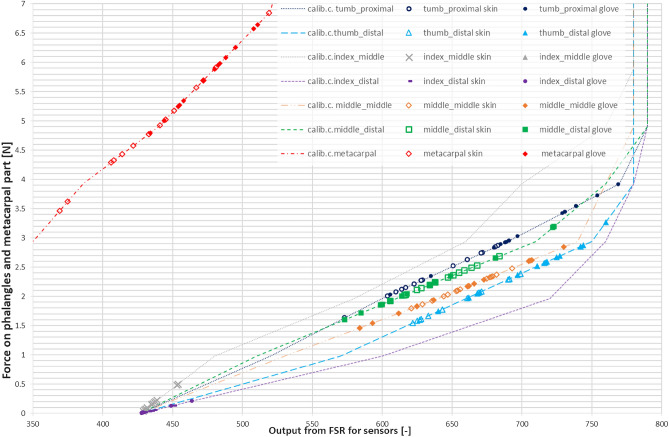
Results of the measurements performed on the selected phalanges and metacarpal part with 1 kg load grabbed with cylindrical grip d = 70 mm.

The resulting weights were recalculated into Newtons and the results have been presented in the graph (Fig. 17). The calibration curves are presented as lines and on top of them, data points from measurements are shown as scatter result series. It was decided to include only the area where the data was obtained, excluding the whole calibration curves range.

The results were subjected to statistical analysis, and they are presented in Table 4. The values given in (brackets) refer to the experiment performed in nitrile glove, whereas the first value is the force in the case of barehand dry grip.

**Table 4:** Statistical data from the measurements performed on the selected phalanges and metacarpal part with 1 kg load grabbed with cylindrical grip (d = 70 mm).

Finger phalange	Average [N]	Median [N]	Max [N]	Min [N]	Stand. Dev [N]
Thumb proximal	2.36/(3.09)	2.27/(2.96)	2.87/(3.91)	1.63/(2.03)	0.34/(0.49)
Thumb distal	1.96/(2.51)	1.97/(2.58)	2.39/(3.27)	1.55/(1.74)	0.31/(0.39)
Index middle	0.18/(0.06)	0.15/(0.06)	0.50/(0.07)	0.04/(0.04)	0.13/(0.01)
Index distal	0.04/(0.03)	0.03/(0.01)	0.14/(0.14)	0.01/(0.01)	0.04/(0.06)
Middle middle	2.15/(2.13)	2.10/(2.18)	2.48/(0.21)	1.79/(1.46)	0.20/(0.40)
Middle distal	2.28/(2.25)	2.24/(2.20)	2.69/(3.20)	1.92/(1.60)	0.21/(0.47)
Metacarpal	4.89/(5.73)	4.78/(5.89)	6.84/(6.65)	3.46/(4.80)	0.85/(0.54)

The first value represents a force for the case of barehand grip and (glove grip) is in brackets.

## Result analysis and discussion

**Figure 18:**
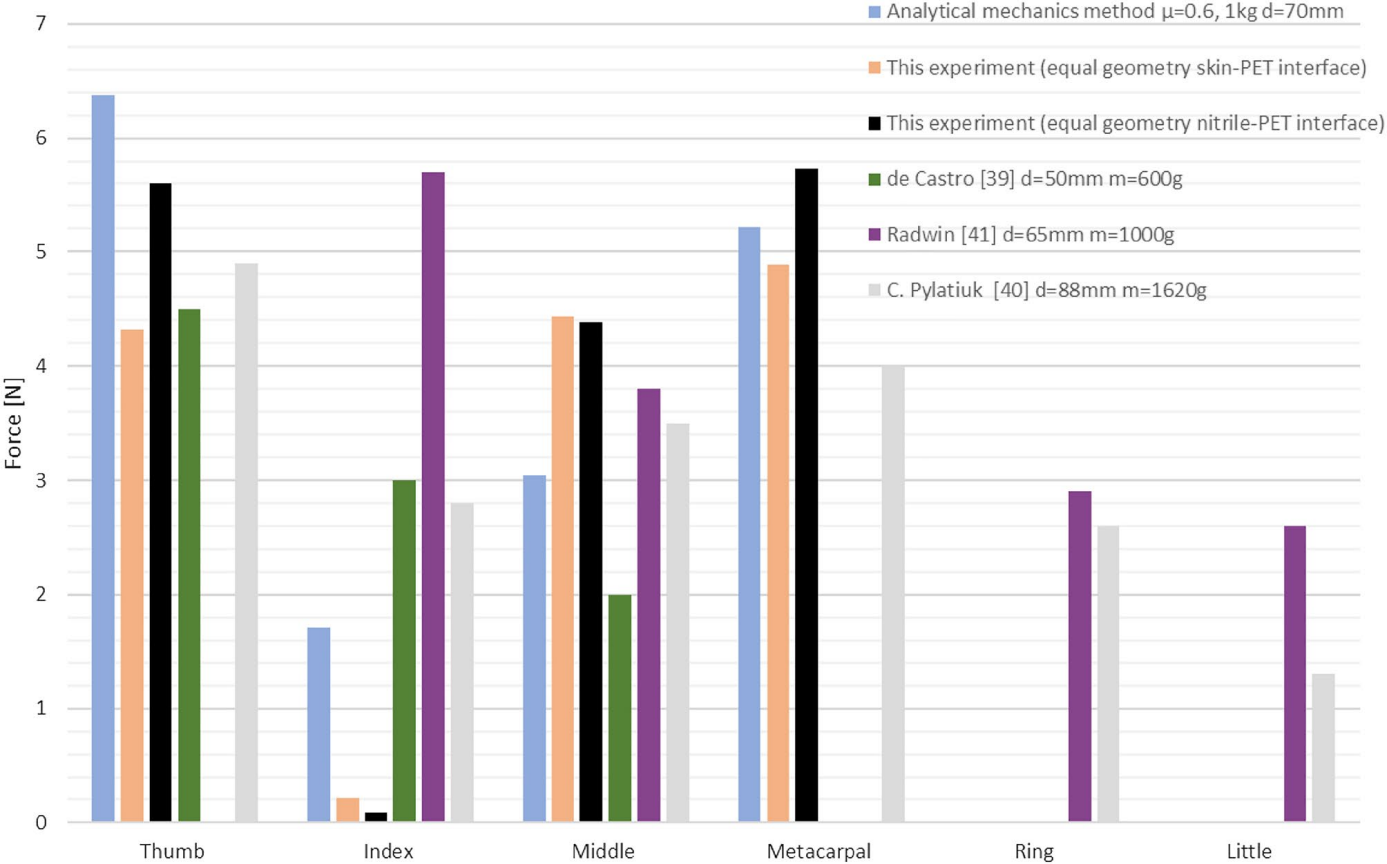
Comparison of forces predicted by the analytical model of cylindrical grip (d = 70 mm, m = 1 kg) with obtained through experimental validation and other studies.

To compare the method and the results from experiment, outcomes from other studies, with subjects, were used. Although the setups were mostly different in terms of geometry and weight, they can still be used to compare e.g., distributions of the forces on the fingers as percentage of the force sum in cylindrical grip. To do this the forces on phalanges were summed by multiplying them times the number of contact points. Although a resultant force should have been used, this approach was consistent with other experiments (Fig 18).

**Figure 19:**
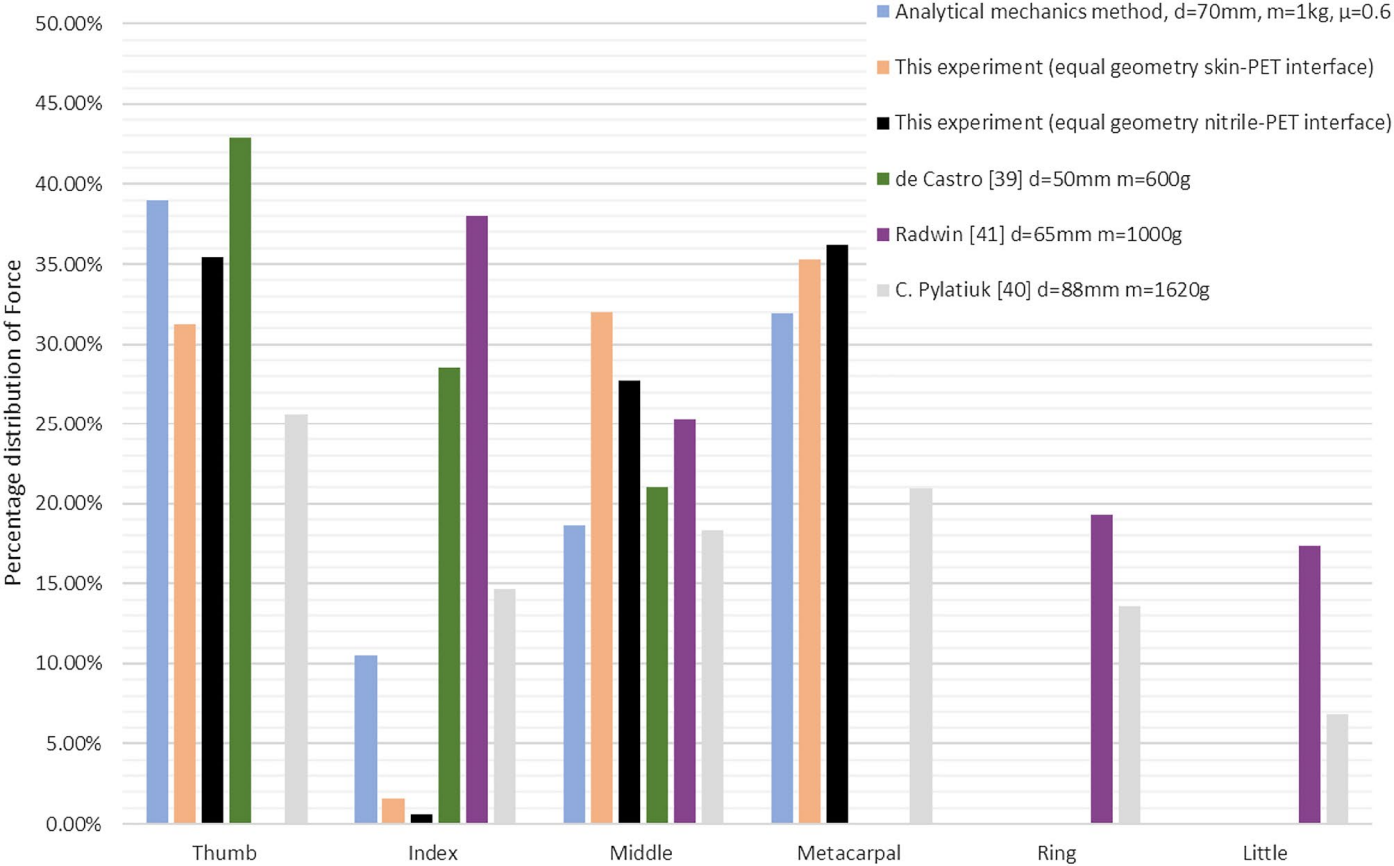
Comparison of distribution of force on load transferring fingers as percentage of the sum of forces in a given method/report.

Obtained results show that the sum of forces predicted with the analytical model is similar to the experiment with equal geometry performed on skin-PET interface and slightly above for nitrile glove to PET interface (16.35N vs. 15.81N and 13.84N respectively). The sum from^39,41^ and^40^ were equal to 9.5N, 15N and 19.1 respectively. Although it is difficult to compare directly, since different loads were used, the force predicted for the thumb was higher than measured and reported in other studies. In case of index finger the prediction falls short of the reported values but is higher than the experiment. This is most likely due to the force acting on middle finger that is applied from the same direction and unloads the index finger in the experiment. Therefore, the middle and index finger should be counted together. The load on metacarpal is similar to experimental value but is higher than the load reported in other study even though it was performed on less weight. The ring and little fingers cannot be compared as they were not included in the analysis. Other compared parameter was percentage distribution of force on fingers in relation to the sum of forces visible in Fig. 19.

The prediction for thumb and middle finger matched the experimental values reasonably, however again it can be noticed that index finger and metacarpal area have different results. Nevertheless, sum of forces suggests that the grip can be performed. The over/underestimation can result from the fact that the angles between phalanges could not be replicated exactly, as there are differences between a real hand and the modeled one. In the model, all phalanges were modeled with equal length and shifted on the metacarpal hand, but in reality, they are obviously not identical. The grip was performed without using all fingers thus forces cannot be directly compared to all of the other studies. In the last case^40^, however, where the metacarpal part was involved as well as all fingers, one can notice that the load is lower on the ring and little finger. Since those fingers usually aren’t driven in the device due to the limited number of actuators, they were omitted.

## Conclusions

The analytical mechanics model developed for cylindrical grip allows for approximation of forces that occur in this grip. As it can be seen in comparison to experiments (both ours and from other studies) the prediction does not match the results exactly, but the obtained forces are enough to lift the cylinder, which was also the objective of the validation. The differences include mostly the index and middle finger since these two act in the same direction and a greater portion of the load could be taken by one of them. It is assumed that the difference comes from the friction coefficients that were assumed and the imperfect reproduction of angles between phalanges. In retrospect, a better idea would be to use elastomer under each finger to reproduce the same contact patch each time instead of putting the fingers directly on the sensors. On the other hand, the real-object element would be lost, and forces applied vertically might be recorded as negative^41^.

The total sum of forces predicted by the method was higher in comparison to a grip with both the glove interface and the barehand grip. From an engineering perspective, a method that overestimates the results increases the safety coefficient of the device, thus it is acceptable. When designing a device using the method, care should be taken to choose materials that have high friction coefficient and can be slightly compliant or deformable to increase the contact area with the given object. Even though the dimensions of the model used in the study might be different from the other projects the method can be reused by inserting other dimensions and angles and solving the equations numerically.

To conclude the relatively simple methodology presented in the work allows to calculate the forces acting on fingers in cylindrical and hook grip for a given model of a hand without necessity to perform the costly experiments or using commercial numerical software. Although, the equations were solved with MATLAB, any other free package could be used such as Wolfram alpha or they could be solved on a calculator. It contributes to the field of hand prosthesis design allowing for estimation of forces necessary in dynamics calculations when choosing e.g. the actuators based on required parameters in the mechatronics model.

Future research could include analytical modelling of other grips and validating them. There exists a handful of experimental studies on this topic, but the solution is mostly valid for the given experiment setup (mass, size, etc.) and interpolated between data points. This allows for approximation of the forces for design purposes, but it is averaged and not case specific. However, from our perspective the methods based on analytical mechanics is also useful, as it consumes less resources, therefore their development is justified. Also, using the methodology to test other designs and observe whether the results are repeatable is warranted (The presented model files are available in Supplementary information 1 and 2).

### Supplementary Information

Supplementary Information 1.

Supplementary Information 2.

## Data Availability

All data generated or analyzed during this study are included in this published article (and its Supplementary Information files). In the case of CAD files, other formats can be provided on request by contacting the corresponding author via e-mail (witold.rzadkowski@pw.edu.pl).
